# HIV and Depression: Examining Medical Students Clinical Skills

**DOI:** 10.3389/fpsyt.2020.00240

**Published:** 2020-03-27

**Authors:** Eliut Rivera-Segarra, Paola Carminelli-Corretjer, Nelson Varas-Díaz, Torsten B. Neilands, Lawrence H. Yang, Guillermo Bernal

**Affiliations:** ^1^School of Behavioral and Brain Sciences, Ponce Health Sciences University, Ponce, Puerto Rico; ^2^Department of Global and Sociocultural Studies, Florida International University, Miami, FL, United States; ^3^Center for AIDS Prevention Studies, University of California at San Francisco, San Francisco, CA, United States; ^4^College of Global Public Health, New York University, New York, NY, United States; ^5^Retired, Río Piedras, Puerto Rico,

**Keywords:** HIV, depression, medical students, clinical skills, standardized patients

## Abstract

Major depression is a prevalent psychiatric disorder among people living with HIV (PWH). Major depression symptoms, including suicidal ideation, can hinder clinical care engagement and anti-retroviral treatment adherence. Research suggests that inquiry about major depression symptomatology and suicidal ideation should be standard practice when offering primary care services to PWH. However, studies examining depression and suicidal ideation inquiry are scarce. This study’s aim was to describe medical students’ clinical skills for dealing with major depression symptomatology and suicidal ideation among PWH in Puerto Rico. A total of 100 4^th^ year medical students participated in a Standardized Patient simulation with a trained actor posing as a PWH and with a previous major depression diagnosis. One-way frequency tables were used to characterize the sample and the percentage of each observed clinical skill. Two key findings stem from these results only 10% of the participants referred the patient to psychological/psychiatric treatment, and only 32% inquired about suicidal ideation. Our findings highlight the need for enhancing medical students’ competencies regarding mental health issues, particularly when providing services to at risk populations such as PWH within primary care settings.

## Introduction

As of 2018, more than 49,000 cumulative cases of HIV/AIDS have been recorded in Puerto Rico since the 1980’s (HIV/AIDS Surveillance Program, Puerto Rico Health Department, 2018). Following the implementation of several prevention programs, recent reports show a slight reduction in the amount new cases diagnosed and recorded per year. However, despite social and public health efforts, an estimated 30% of PWHA in Puerto Rico remain outside the pipeline of care and lack necessary medical treatment (HIV/AIDS Surveillance Program, Puerto Rico Health Department, 2017).

Major depressive disorder (MDD) continues to be the main psychiatric diagnosis among people living with HIV (PWH) (1, 2). The prevalence of MDD among PWH has been estimated at 65% in the US and Puerto Rico (3). MDD is generally characterized by decreased interest and pleasure in daily activities, changes in sleep patterns and appetite, feelings of guilt or worthlessness, inability to concentrate, and impaired social functioning present during at least 2 weeks (4). Therefore, it can have negative implications for individuals already dealing with a challenging chronic disease. In extreme cases of MDD, suicidal ideations and behaviors are other major symptoms that can often be present. Suicide behaviors are conceptualized as a continuum of acts ranging from suicidal thoughts or suicidal ideations, to intended self-harm or suicide attempts (5). Research has identified suicide behaviors to be more prevalent among PWH than the general population (2, 5). In Puerto Rico, an estimated prevalence of 22% of suicide behaviors has been documented among PWH, almost four times higher than the general population (6).

Research has documented that symptoms of MDD, including suicide behaviors, can negatively impact PWH by hindering clinical care engagement and anti-retroviral treatment adherence (7). In addition, literature has also documented that PWH and MDD are more likely to experience suppressed viral loads, lower CD4 cell counts, substance abuse, and increased anxiety symptoms (8, 9). Thus, addressing MDD symptomatology among PWH should be an integral part of their medical HIV treatment and retention in the continuum of care (8, 10).

Fortunately, evidence-based treatments for MDD and suicide behaviors have shown to be efficacious for PWH (11). Research has documented that the alleviation of MDD symptoms is associated with better medical treatment outcomes including: anti-retroviral treatment adherence, appointment attendance after 12 months, and lower mortality odds (12–14). However, a recent study found that less than 15% of PWH and MDD received evidence-based treatment for depressive symptomatology (15). This percentage is even lower for ethnic minorities (e.g., Latinos) living with HIV and MDD as they are more likely to experience suicide behaviors but less likely to engage in evidence-based treatment for MDD (15, 16). In addition, research has evidenced that instead of seeking specialized treatment, people living with a psychiatric disorder tend to seek help by visiting their primary care physician (17). Also, more than half of suicide victims contact their primary care physicians at least 4 weeks prior to their deaths (18). Therefore, primary care clinics are an ideal setting for identification and early treatment of MDD symptomatology and suicide behavior.

Research suggests that inquiry of MDD and suicidal ideation should be standard components of primary care for PWH (5). However, few research efforts have examined primary care physicians’ clinical proficiency in assessing and diagnosing psychiatric illnesses *via* the direct observation of patient/provider interactions. Unfortunately, the scarce published research presents a worrisome scenario documenting that physicians often lack the necessary skills to detect and manage psychiatric diagnoses (19). One study using actors as standardized patients in the U.S. found that only 36% of physicians actively inquired about suicide behaviors during clinical interactions (20). Still, studies examining how primary care physicians address MDD and suicidal ideation symptomatology among ethnic minorities living with HIV are scarce. This gap in the research literature highlights the need to understand how symptoms MDD including suicidal ideation are managed during clinical interactions with ethnic minorities living with HIV. Therefore, our study aimed to document medical students’ clinical skills for recognizing and managing symptoms of MDD and suicidal ideation among PWH in Puerto Rico.

## Method

This research study is part of a larger project detailed elsewhere, (21) which aimed to examine HIV stigma behaviors during clinical interactions. This research study was approved by the IRBs at the University of Puerto Rico and the Florida International University. For the purposes of the current study, we implemented Standardized Patient (SP) simulations and observational techniques.

We engaged 4^th^ year medical students in SP simulations of clinical cases that were previously designed by our team and then validated by a focus groups of PWH and the University of Puerto Rico’s SP Program. In SP simulations, participants are exposed to SP cases presented by trained actors in order to simulate what physicians encounter in actual clinical scenarios. SP simulations are used widely, both domestically and internationally, as a tool for training physicians given they enable realistic reenactments of clinical interactions under controlled conditions. They are used to evaluate students’ medical knowledge, as well as communication and interpersonal skills. Some of the most commonly evaluated medical skills include: a) interview skills for documenting medical history, b) physical examination protocols, c) interpretation of medical results for patients, and d) patient orientation (22). Medical programs using SP simulations have documented the impact on provider performance, showing improved communication and cultural competence skills that can be sustained over time (23). SP simulations are a promising interdisciplinary technique for health related research. Unfortunately, SP simulation techniques have not been integrated widely into HIV research. Below we provide a detailed description of our research method.

### Participants

The sample consisted of one hundred (N = 100) 4^th^ year medical students in Puerto Rico who engaged in SP simulations with trained actors portraying the role of a PWH and previously diagnosed MDD in a primary care context. Participants were recruited at a medical school during routinely SP simulation evaluations during their 4^th^ year of training, which are used as a standard assessment technique in medical schools in Puerto Rico. Each year, medical students must complete SP simulation evaluations during which they engage in approximately eight different clinical cases. Participants met the following eligibility criteria: 1) had the legal age to consent in Puerto Rico (21 years of age), 2) had completed their fourth year of medical school training and were ready to begin their residency, and 3) self-identified as Puerto Rican. We selected participants who had completed their fourth and last year of medical training as they are closer to becoming a practicing physician. By this time students have had vast experiences with SP and real clinical scenarios, and should possess enough medical knowledge, training, and experience related to basic infectious diseases and mental health illnesses. The final sample was almost equally divided by gender (53% female), most were single (78%), and self-identified as Catholic (44%). A more detailed information on the sample’s demographic characteristics is presented in **Table 1**.

**Table 1:** Socio-demographic characteristics.

Variable	N	%
Age		
21–25	35	35.0
26–30	55	55.0
31–35	6	6.0
36–40	2	2.0
41>	1	1.0
Gender		
Male	46	46.5
Female	53	53.5
National origin		
Puerto Rican	100	100.0
Racial origin		
White	60	60.6
African-American	4	4.0
Native American	1	1.0
More than one race	34	34.3
Civil status		
Married	11	11.0
Single	78	78.0
Divorced	1	1.0
Living with a partner	8	8.0
Other	2	2.0
Religious affiliation		
Catholic	44	44.4
Protestant	13	13.1
Jehovah Witness	1	1.0
Episcopal	1	1.0
None	34	34.3
Other	6	6.1
Religion importance		
Not important	29	29.3
Somewhat important	29	29.3
Important	22	22.2
Very important	19	19.2
Religious activities		
Do not participate	57	57.6
Weekly	12	12.1
Several times a month	9	9.1
Several times a year	21	21.2
Annual income		
<$10,000	25	25.0
$10,001–$20,000	6	6.0
$20,001–$30,000	7	7.0
$30,001–$40,000	9	9.0
$40,001–$50,000	10	10.0
$50,001–$60,000	9	9.0
>$60,000	34	34.0
Employment status		
Employed (Part-time)	1	1.0
Employed (Full-time)	4	4.0
Not employed	95	95.0

Percentages and N’s will not always sum 100% due to small amounts of missing data.

### Procedure

During routine SP simulation evaluations, participants engaged in a SP simulation with a trained actor posing as a patient living with HIV and previously diagnosed MDD in a primary care context. This experimental condition was randomized and interspersed amidst other non-HIV SP simulations included in the medical school’s evaluation to avoid them becoming saturated with HIV and MDD cases and influencing their behaviors in the study. All patients initially reported a uniform health concern (symptoms of a common cold) as part of their chief complaint. During the SP simulation, the trained actors were required to do the following: 1) uniformly present the symptoms of a common cold, 2) reveal their HIV positive status and previous MDD diagnosis during the course of the medical history, 3) inform using medication to treat MDD, 4) inform psychological treatment discontinuation, 5) deny suicidal ideation or intent if inquired, and 6) request follow-up instructions on how to treat their symptoms. Three trained actors engaged in the study to ensure there were sufficient personnel even in the case of actor dropouts or sickness. All actors were trained by the University of Puerto Rico’s SP program and had completed 200 or more hours of SP simulated interactions. They were trained using the specific protocols for this study in collaboration with physicians to ensure accuracy when performing the developed cases.

The SP Program staff informed 4^th^ year students of our study. Those who wished to participate were offered an orientation about the study by the research team and the SP staff. Once potential participants agreed to engage in the study and signed the consent form, they completed the demographic questionnaire and proceeded to engage in their SP simulations. They were provided with a mock patient file with a uniform profile (i.e., age, gender). Each encounter was video-recorded for subsequent analysis. The SP Program had three ceiling mounted cameras that recorded interactions from multiple angles in order to have a clear perspective of the physician and the trained actor simultaneously.

Because participants were aware that they would engage in an observed simulation, some social desirability could be manifested. To address this concern, they were not told which of the actors in the evaluation would reveal they were living with HIV and MDD before engaging in the SP simulation. We implemented this step to ameliorate the effects of social desirability while not eliminating it completely, as some level of social desirability is unavoidable in direct observation studies.

### Instruments

Demographic Questionnaire—This 20-item questionnaire addressed participants’ income, age, gender, sexual orientation, ethnicity, and religious affiliation, among other variables.

Mental Health Skills Inventory (MHSI)—This inventory was developed by our team, in collaboration with our consultants with expertise in SP-based measures to assess medical students’ clinical skills when interacting with a patient presenting a mental health issue (i.e. MDD symptomatology). The MHSI includes nine clinical skills associated to mental health proficiency that the literature documents as important for patient/provider engagement and inclusion of patients into the pipeline of care (i.e. referring the patient to psychiatric/psychological treatment, exploring mental health history). **Table 2** includes the complete list of clinical skills for the MHSI. All clinical skills are assessed using a three-point scale with the following values: manifested (2), unsure if manifested (1), and not manifested (0). The MHSI was reviewed by a panel of experts on HIV and mental health research to establish its content validity. We have successfully used this technique to validate observational measures for SP simulations with socially stigmatized health conditions (21, 24, 25). Three experts on HIV related research, observational research techniques, and SP protocol development used the inventory to code five previously recorded interactions using our developed SP case. The coding between the experts was compared, divergent results were discussed, and the items were refined as needed. This video coding and item refinement process was implemented on three occasions until 100% inter-rater consensus was reached on the observed behaviors and their coding.

**Table 2:** Physician/Patient Interaction Behaviors (N = 100).

Variable	Manifested		
	N	%	95% CI
Psychiatric Related Behaviors			
**Screening for depression**			
Asked further about the depression diagnosis.	44	44%	34%, 54%
Explored mental health history.	14	14%	8%, 22%
Explored neurovegetative symptoms of depression (i.e. sleep, diet, concentration).	38	38%	28%, 48%
**Treatment abandonment**			
Asked the reasons for abandoning depression treatment.	2	2%	0.2%, 7%
Asked if the medication currently being used was prescribed.	23	23%	15%, 32%
**Comorbidity**			
Offered the patient information about the relationship between depression and poor HIV outcomes (i.e. viral load impact, CD4 count, etc.).	4	4%	1%, 10%
**Suicide ideation**			
Asked about suicide ideation or intent (current or previous).	32	32%	23%, 42%
**Referral to care**			
Talked about the importance of receiving psychiatric/psychological treatment.	28	28%	19%, 38%
Referred the patient to psychiatric/psychological treatment.	10	10%	4%, 16%

95% confidence intervals are exact (Clopper-Pearson) intervals.

### Data Analysis

Given the descriptive research goals of this study, one-way frequency tables were used to characterize the sample and the percentage of each observed mental health related clinical skill (i.e. depression/suicide inquiry or psychiatric referral). To estimate intervals surrounding percentages, we computed 95% exact Clopper-Pearson confidence intervals.

## Results

The documented clinical skills exhibited by participants during SP interactions evidence a concerning scenario for potential clients. Below we provide the frequency of manifested clinical skills related to: screening, treatment abandonment, comorbidity, suicide ideation, and referral to care.

Screening for MDD—Our results evidence that 56% did not ask the patient if they had been previously diagnosed with MDD, nor inquired about MDD symptomatology, even though the patient mentioned feeling depressed. More concerning, 86% did not explore or asked questions about the patient’s mental health history.

Treatment abandonment—Abandoning medical care treatment is common among patients living with MDD. The patient actors were trained to state during the interaction that they had abandoned psychological/psychiatric treatment for MDD despite continued use of non-prescribed medication obtained from a friend. Almost all participants (98%) neglected to ask patients about the reasons for abandoning treatment.

Comorbidity—Despite the high comorbidity of mental health conditions among PWH, only 38% of participants informed patients about the influence of untreated MDD on their HIV treatment outcomes.

Suicidal ideation—When MDD goes untreated and its symptoms become severe, the potential for suicidal behaviors increases. During our SP simulations only 32% of the sample explored suicidal ideation.

Referral to care—Primary care physicians are in an advantageous position to refer patients to specialized services, including treatment for mental health. Most participants did not talk with patients about the importance of engaging in care for MDD (72%) and neglected to refer the patient for psychological treatment (90%).

## Discussion

The findings from this study suggest that participants in this context lacked some of the necessary clinical skills to manage mental health issues among PWH. These results are consistent with findings from previous studies documenting physicians’ less than acceptable clinical skills to assess MDD and suicidal ideation during primary care interventions (20, 26). This lack of clinical skills may be even more damaging when patients present other comorbid chronic health conditions such as HIV. The training of future physicians on clinical skills to address mental health issues should be a priority in order to successfully integrate mental health care into traditional HIV medical care. Some of this training should begin early during their medical school years, as once in residency it is less likely that they will receive focused and substantive training on the recognition and treatment of mental health comorbidities. Furthermore, the use of simulation methods such as SP could be a useful strategy as it has been recently documented as effective in assisting with mental health education (27).

Two key findings that stem from these results merit particular attention. In our study, only 10% of the patient actors were referred to psychological/psychiatric treatment following the SP intervention. In addition, only 32% of medical students inquired about suicide behaviors during their interactions. Both findings are lower than what has been reported in the few previous studies with physicians in real life scenarios (20, 28), suggesting that the lack of mental health clinical skills among physicians is present from early on during their medical training and could persist after training is completed. This points towards a need to address medical students’ mental health clinical proficiency early on during their training. Scientific literature suggests that the lack of clinical skills associated to mental health diagnoses among physicians could be explained by individual styles of interacting with patients (20). Although our study did not address this issue results suggest that other important factors, such as medical training, are at play and may surpass individual level considerations. Perhaps a medical training solely framed upon a biomedical model, that focuses on treating physical health conditions as an only priority (e.g. HIV), could explain medical students’ lack of attention to presented mental health issues; which would also be congruent with a recent study suggesting that psychosocial clinicians had better mental health skills than biomedical clinicians (26).

This study design has some limitations. Firstly, participants were aware that SP were not actual patients, which could have prompt them to modify behaviors. Secondly, participants were aware they were being observed which could have also influenced stigmatizing behaviors that would have been more salient in a real clinical interaction. Thirdly, individual styles of interactions and characteristics (i.e. lower tone of voice or social class) were not addressed in the current study and should be addressed by future research. Finally, we did not include hypothesis testing because, to the best of our knowledge, no guideline specifies the desired frequency of behaviors for a patient history such as the one presented in our SPS. Thus, our results should only be interpreted in light of the previous cited studies on the topic.

In summary, despite the limitations, our findings highlight the importance of enhancing medical students’ competencies in managing mental health issues, particularly when providing services to PWH in the context of primary care. Medical schools are an ideal scenario to develop and implement such training in order to foster the knowledge and clinical skills needed by medical students in Puerto Rico. This is a vital first step in order to address the needs of Latino PWH and MDD.

## Data Availability Statement

All datasets generated for this study are included in the article.

## Ethics Statement

IRB committees from the University of Puerto Rico and the Florida International University approved the study.

## Author Contributions

ER-S, PC-C, and NV-D: study design, data collection, data analysis, writing, and editing of manuscript. TN: study design, data analysis, and editing of manuscript. LY and GB: writing and editing of manuscript.

## Funding

This research was funded by the National Institute on Drug Abuse (1K02DA035122). ER-S and this publication were supported by the National Institutes on Minority Health and Health Disparities under awards U54MD007579 and G12MD007579 and the American Psychological Association (APA; ProDIGS). This article does not represent the opinion of the National Institutes of Health or APA.

## Conflict of Interest

The authors declare that the research was conducted in the absence of any commercial or financial relationships that could be construed as a potential conflict of interest.
